# Localization of genes involved in the metabolic syndrome using multivariate linkage analysis

**DOI:** 10.1186/1471-2156-4-S1-S57

**Published:** 2003-12-31

**Authors:** Curtis Olswold, Mariza de Andrade

**Affiliations:** 1 Division of Biostatistics, Department of Health Sciences Research, Mayo Clinic, Rochester, Minnesota, USA

## Abstract

There are no well accepted criteria for the diagnosis of the metabolic syndrome. However, the metabolic syndrome is identified clinically by the presence of three or more of these five variables: larger waist circumference, higher triglyceride levels, lower HDL-cholesterol concentrations, hypertension, and impaired fasting glucose. We use sets of two or three variables, which are available in the Framingham Heart Study data set, to localize genes responsible for this syndrome using multivariate quantitative linkage analysis. This analysis demonstrates the applicability of using multivariate linkage analysis and how its use increases the power to detect linkage when genes are involved in the same disease mechanism.

## Background

It has been shown that for correlated traits, multivariate approaches for genetic linkage analyses can increase the power and precision to identify genetic effects [1-4]. When correlated measures are considered, the composite score from joint consideration of all measures reflects a smaller level of measurement error than each of the univariate measures [5]. Then, multivariate analysis provides a statistically efficient mechanism for controlling the analysis-wise significance level when there are multiple trait observations for each subject [3,6]. Therefore, using methods that can analyze several traits jointly is likely to enhance the ability to identify genes influencing the metabolic syndrome. Although multivariate Haseman-Elston (H-E) [7] and variance-components (VC) methods [8] have been available for several years, only recently has the power of these methods been compared. Allison et al. [6] presented results from a large simulation study to assess the effectiveness of a bivariate H-E test for linkage versus the univariate H-E test [9]. Their results showed that bivariate analyses can improve the power to detect linkage, with a greater gain in power when the genetic covariance due to a major locus linked to the marker studied is negative and the residual covariance among the traits is positive. Amos et al. [3] also showed that bivariate approaches are more powerful than univariate analyses except for traits with very high positive polygenic correlation. Evans [4] also reached similar conclusion.

Our approach is based on the assumption that it is easier to detect a quantitative trait locus (QTL) involved in the metabolic syndrome using multivariate linkage analysis. Our aim is to show that using combinations of traits related to the metabolic syndrome, and then using them in multivariate linkage analysis software, gives reliable results for linkage to genes associated with this syndrome.

## Methods

### The metabolic syndrome

There are no well accepted criteria for the diagnosis of the metabolic syndrome. However, the metabolic syndrome is identified by the presence of three or more of the variables listed in Table 1[10].

### Multivariate linkage analysis

The multivariate variance-components (MVC) approach is an extension of the univariate approach described by Amos [8]. For multivariate traits, let **Y_*i *_**= (Y_11_,...,Y_1*ki*_,...,Y_*mki*_)' be a vector of *m *multivariate trait values for *k*_*i *_members of the *i*^th ^family. Let *N *be the total number of families, β a vector of dimension *mp *of the regression coefficients for the *p *covariates (including a vector of **1**'s corresponding to the overall mean), **X**_*i *_= **I**_*m*_**X**_*ki x m *_an *mk*_*i *_× *mp *known matrix of covariate values for the *i*^th ^family, where  is the Kronecker product, and **V_*i *_**a VC matrix of dimension *mk*_*i*_× *mk*_*i*_. Then, the variance-covariance matrix of the traits is **V_*i *_= A****G_*i *_+ B****Z_*i *_+ C****I_*i*_**, where **G_*i *_**is the *k*_*i *_× *k*_*i *_matrix of the coefficients of relationship for the family *i*; **Z_*i *_**an *k*_*i *_× *k*_*i *_matrix of estimated proportion of alleles identical by decent (IBD) for pairs of related individuals for the *i*^th ^pedigree; **I_*i *_**is the *k*_*i *_× *k*_*i *_identity matrix; and **A, B**, and **C**, are, respectively, polygenic, major-gene, and environment variance-covariance matrices each of dimension *m *× *m*. A more detailed description of these models was presented elsewhere [11,12].

### Multivariate VC test

To test for genetic linkage, we also construct a likelihood ratio test. Under the null hypothesis, the major gene parameter(s) are restricted to equal **0**. The distribution of the multivariate test is a mixture of χ^2 ^values [13]. For trivariate linkage analysis of an additive genetic effect, the distribution of the trivariate test that the major-gene covariance components are zero is a mixture of 1/8 χ_0_^2^, 3/8 χ_1_^2^, 3/8 χ_3_^2 ^and 1/8 χ_6_^2^. One-eighth of the time all the VCs are estimated to be positive with all the covariances different from 0 yielding 6 degrees of freedom. Three-eighths of the time, one of the VCs is estimated to be zero with two covariances fixed to zero (yielding 3 degrees of freedom). Another three-eighths of the time two VCs are fixed to zero with all covariances equal to zero yielding 1 degree of freedom. Finally, one-eighth of the time all the variances are fixed to zero resulting in a degenerate distribution of point mass at zero.

For the multivariate linkage analysis, we use the following four traits: triglycerides, HDL-cholesterol, systolic blood pressure (SBP), and fasting glucose. Since these variables, except for triglycerides, were measured at several time points, we applied a similar regression approach described in Levy et al. [14] for these four variables and then used their residuals as the quantitative traits in the multivariate genome-wide linkage analysis for quantitative traits. There are two packages that use the MVC approach: ACT [15] and EMVC [16]. The analyses here presented were performed using the EMVC package using 330 families with 4692 individuals, of whom 1702 have genotype information.

## Results

We do observe small to moderate positive genetic correlations between SBP and triglycerides (0.187), SBP and fasting glucose (0.296), and triglycerides and fasting glucose (0.361); we also observe a strong negative correlation between HDL-cholesterol and triglycerides (-0.664), and small to moderate negative correlations between HDL-cholesterol and SBP (-0.048), and HDL-cholesterol and fasting glucose (-0.249). Table 2 shows the pair-wise polygenic and the quantitative trait locus (qtl) correlation among the four traits at the position where evidence for linkage was found for the trivariate linkage analysis. We observed moderate to strong polygenic and qtl correlation for all traits except for polygenic correlation for SBP and fasting glucose SBP and HDL-cholesterol on chromosome 6 at 152 cM.

**Figure 1:**
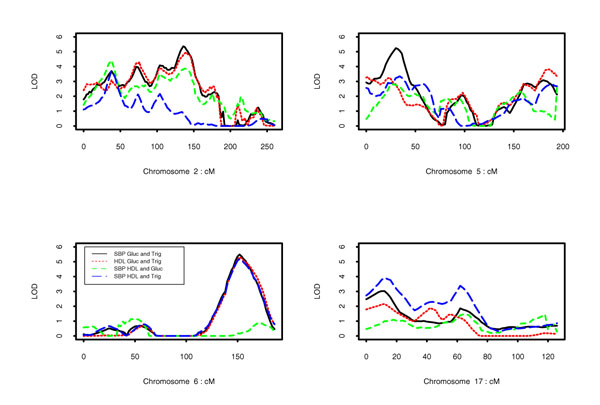
**Comparison of LOD profiles for trivariate multipoint linkage analyses on chromosomes 2, 5, 6, and 17 **The black line represents the trivariate traits SBP, glucose, and triglycerides. The red dot line represents the trivariate traits HDL, glucose, and triglycerides. The green line represents the trivariate traits SBP, HDL, and glucose. The blue line represent the trivariate traits SBP, HDL, and triglycerides.

Figure 1 depicts the trivariate multipoint linkage analyses results of chromosomes 2, 5, 6, and 17. Because of space constraints we show only the trivariate results. The trivariate lod scores were obtained using EMVC program [16]. On chromosome 2, the following combination produced evidence for linkage: SBP, fasting glucose, and triglycerides (LOD  5.37, position 136 cM, *P *= 5.4 × 10^-5^); HDL, fasting glucose, and triglycerides (LOD  4.97, position 140 cM, *P *= 1.7 × 10^-4^); SBP, HDL, and fasting glucose (LOD  4.42, position 38 cM, *P *= 5 × 10^-4^); SBP, HDL, and triglycerides (LOD  3.70, position 38 cM, *P *= 1.5 × 10^-3^). The univariate maximum LOD scores for SBP, triglycerides, fasting glucose, and HDL were, respectively, 1.5 (34 cM), 1.75 (74 cM), 3.3 (136 cM), and 1.2 (38 cM). On chromosome 5, the following combination produced evidence for linkage: SBP, fasting glucose, and triglycerides (LOD  5.24, position 30 cM, *P *= 7 × 10^-5^); HDL, fasting glucose, and triglycerides (LOD  3.81, position 186 cM, *P *= 1.2 × 10^-3^); SBP, HDL, and triglycerides (LOD  3.35, position 34 cM, *P *= 2.8 × 10^-3^); SBP, HDL, and fasting glucose (LOD  2.80, position 30 cM, *P *= 7.9 × 10^-3^). The univariate maximum LOD scores for SBP, triglycerides, fasting glucose, and HDL were, respectively, 2.21 (34 cM), 1.97 (0 cM), 1.53 (160 cM), and 0.16 (160 cM). On chromosome 6, the following combination produced evidence for linkage: SBP, fasting glucose, and triglycerides (LOD  5.49, position 152 cM, *P *= 5 × 10^-5^); HDL, fasting glucose, and triglycerides (LOD  5.30, position 152 cM, *P *= 6 × 10^-5^); SBP, HDL, and triglycerides (LOD  5.18, position 152 cM, *P *= 1 × 10^-4^). The univariate maximum LOD scores for SBP, triglycerides, fasting glucose, and HDL were, respectively, 0.12 (2 cM), 5.52 (152 cM), 0.64 (44 cM), and 0.25 (182 cM). On chromosome 17, the following combination produced evidence for linkage: SBP, fasting glucose, and triglycerides (LOD  3.02, position 10 cM, *P *= 5.2 × 10^-3^); SBP, HDL, and triglycerides (LOD  3.91, position 12 cM, *P *= 1.2 × 10^-3^). The univariate maximum LOD scores for SBP, triglycerides, fasting glucose, and HDL were, respectively, 1.35 (66 cM), 1.76 (6 cM), 0 (-), and 0.22 (126 cM).

## Discussion

The MVC approach appears to perform well in the identification of regions linked to genes associated with traits related to the metabolic syndrome, mainly on regions where the QTL effects were negatively correlated and there was a positively correlated polygenic effect as shown by Amos et al. [3] and Evans [4]. Our results did identify a minor linkage peak to the same region of chromosome 17 described by Levy et al. [14]. The only region on chromosome 17 using the trivariate VC approach that showed evidence for linkage was on the surrounding region of 10 cM, which was due primarily to the bivariate combination, SBP and triglycerides, (LOD  3.14, position 12 cM, results not shown). Furthermore, evidence for linkage was also found on chromosomes 2, 5, and 6. We also showed that the pair-wise combinations with evidence for linkage are the ones that have either small to moderate genetic correlation or negative genetic correlation. In summary, the use of multivariate quantitative trait loci linkage analysis can increase the power to detect a QTL. However, this procedure is computationally intensive, i.e., the CPU time increases exponentially as the number of traits increases additively.

## Figures and Tables

**Table 1:** Clinical identification of the metabolic syndrome

Risk Factor	Defining Level
Abdominal Obesity	Waist Circumference
Men	> 102 cm (> 40 in)
Women	> 88 cm (> 35 in)
Triglycerides	= 150 mg/dL
HDL cholesterol	
Men	< 40 mg/dL
Women	< 50 mg/dL
Blood Pressure	= 130/85 mm Hg
Fasting Glucose	110–125 mg/dL

**Table 2:** Values of polygenic and QTL correlation between the variables involved in the metabolic syndrome at different locations

				QTL Effects for Traits			Polygenic Effects for Traits		
				
Traits^A ^(1, 2, 3)	Chr	Pos (cM)	LOD	1 and 2^B^	1 and 3	2 and 3	1 and 2^C^	1 and 3	2 and 3
S,G,T	2	136	5.37	0.409	-0.62	0.342	0.62	0.404	0.873
H,G,T	2	140	4.97	-0.81	-0.83	0.573	-0.18	-0.64	0.858
S,H,G	2	38	4.42	0.557	0.25	-0.21	-0.54	0.565	-0.53
S,H,T	2	38	3.70	0.637	-0.32	-0.19	-0.57	0.592	-0.82
S,G,T	5	30	5.24	0.317	0.096	0.954	0.122	0.525	-0.58
H,G,T	5	186	3.81	0.078	0.683	0.122	-0.7	-0.97	0.802
S,H,G	5	30	2.80	0.602	0.138	0.235	-0.27	0.721	-0.59
S,H,T	5	34	3.35	-0.14	-0.15	0.149	-0.06	0.643	-0.74
S,G,T	6	152	5.49	0.084	0.405	0.799	0.32	0.289	0.69
H,G,T	6	152	5.30	-0.03	-0.89	0.365	-0.27	-0.84	0.59
S,H,T	6	152	5.18	-0.37	0.33	-0.91	0.003	0.19	-0.87
S,G,T	17	10	3.02	0.477	-0.33	0.308	0.331	0.727	0.44
S,H,T	17	12	3.91	0.694	-0.34	-0.08	-0.35	0.622	-0.90

^A^S, systolic blood pressure; G, fasting glucose; T, triglycerides; H, HDL-cholesterol.
